# Kv3.3 Expression Enhanced by a Novel Variant in the Kozak Sequence of *KCNC3*

**DOI:** 10.3390/ijms252212444

**Published:** 2024-11-20

**Authors:** Marlen Colleen Reis, Frauke Härtel, Antje Maria Richter, Michaela Weiß, Lea-Theresa Mösle, Reinhard Heinrich Dammann, Dagmar Nolte

**Affiliations:** 1 Institute of Human Genetics, Department of Medicine, Justus Liebig University Giessen, 35390 Giessen, Germany; marlen.reis@humangenetik.med.uni-giessen.de (M.C.R.);; 2 Institute of Physiology, Justus Liebig University Giessen, 35390 Giessen, Germany; 3 Institute of Genetics, Department of Biology, Justus Liebig University Giessen, 35390 Giessen, Germany; antje.m.richter@gen.bio.uni-giessen.de (A.M.R.); reinhard.dammann@gen.bio.uni-giessen.de (R.H.D.); 4 Innere Medizinische Klinik II, Klinikum Memmingen, 87700 Memmingen, Germany

**Keywords:** Kozak sequence, 5’-UTR, ataxia, SCA13, *KCNC3*, Kv3.3

## Abstract

Pathogenic variants in *KCNC3*, which encodes the voltage-gated potassium channel Kv3.3, are associated with spinocerebellar ataxia type 13. SCA13 is a neurodegenerative disease characterized by ataxia, dysarthria and oculomotor dysfunction, often in combination with other signs and symptoms such as cognitive impairment. Known disease-causing variants are localized in the protein coding regions and predominantly in the transmembrane and voltage sensing domains. In a patient with an ataxic movement disorder and progressive cognitive decline, the c.-6C>A variant was detected in the Kozak sequence of *KCNC3*. The Kozak sequence is responsible for efficient initiation of translation. Functional analysis of the new c.-6C>A variant and the upstream 5’-UTR region of *KCNC3* by luciferase assays, quantitative PCR and methylation analysis shows increased protein expression but no effect on transcription rate. Therefore, increased translation initiation of *KCNC3* transcripts compared to wild-type Kozak sequence seems to be the cause of the increased expression. Variants in the regulatory elements of disease-causing genes probably play an underestimated role.

## 1. Introduction

Pathogenic variants in the coding sequence of a gene, such as base substitutions, insertions, deletions and repeat expansions, are known to cause altered mRNA stability, loss of coding sequence leading to truncated proteins, and altered function of the derived proteins, among others. Impairment of transcription and translation efficiency can be caused by cis-elements in the corresponding genes. Focusing on the 5′-untranslated region (5′-UTR) of a gene, such elements include core promoter regions such as the TATA- and the CAAT-box, or gene-specific promoter and enhancer elements [1]. Furthermore, changes in the methylation status of a CpG site or island can affect transcription by altering chromatin accessibility and transcription factor binding [2,3].

Translation efficiency in turn depends on the Kozak sequence, a conserved cis-element containing the translation start codon ATG in the genomic context, or AUG in the transcript, which facilitates proper ribosome binding [4]. The established consensus Kozak sequence is GCCRCCAUGG, but variants of this sequence are also known and functional [4,5]. Mutations of the core Kozak sequence often result in impaired translation and reduced protein expression [1,6,7]. In rarer cases, translation efficiency is increased, but also pathogenic [8,9]. The translational efficiency of a transcript can be affected not only by changes in the Kozak sequence, but also by short upstream open reading frames (uORFs) that can occur in the 5′ non-coding region [10].

Pathogenic variants in the 5′ regulatory regions of genes have been associated with several examples of human disease. However, they have rarely been described in neurodegenerative diseases such as spinocerebellar ataxias (SCAs). SCAs are a clinically and genetically heterogeneous group of autosomal dominant inherited neurodegenerative diseases [11]. SCA type 13 (SCA13; OMIM #605259) is clinically characterized by a combination of gait or stance ataxia, dysarthria, and oculomotor dysfunction. Additional signs and symptoms, such as cognitive decline, tremor and seizures occur in some of the described families [12]. SCA13 is associated with pathogenic, protein-coding variants in the *KCNC3* gene [13], which encodes the voltage-gated potassium channel Kv3.3 [14]. These variants cause amino acid substitutions in Kv3.3, some of which have been shown to alter channel expression and proper channel function [13,15,16,17,18,19,20,21,22,23].

Here, a novel 5′ non-coding variant was detected in the Kozak sequence of *KCNC3*. This variant was analyzed at both the transcriptional and post-transcriptional levels to investigate its effect on gene expression.

## 2. Results

### 2.1. Clinical Findings

**Figure 1:**
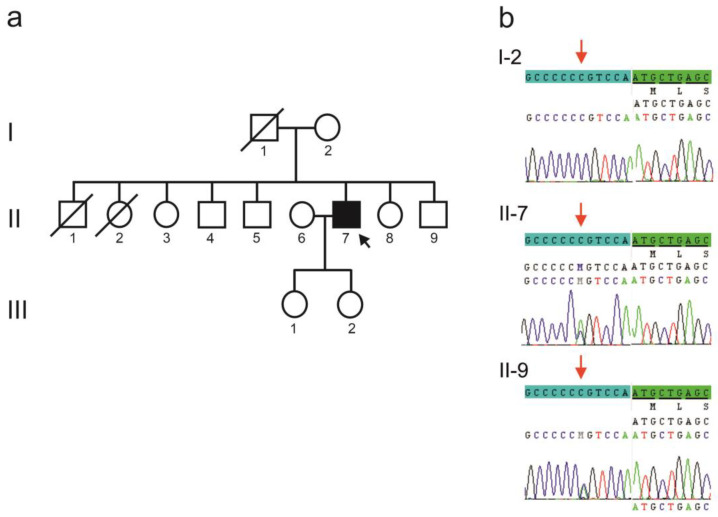
(**a**) Pedigree of a family with a spinocerebellar ataxia syndrome in one patient (arrow). (**b**) Electropherograms of *KCNC3* sequencing in the index patient (II-7), his brother (II-9), and their mother (I-2). A red arrow indicates the c.-6C>A change. The untranslated sequence is highlighted in light blue and the translated sequence is highlighted in green.

The index patient (Figure 1a, II-7) came to clinical attention at age 40. He was diagnosed with an initially slowly progressive ataxic syndrome of unknown etiology, which, according to the patient, began at age 38. The patient’s speech was dysarthric, and ambulation was possible with the aid of a walker. An MRI scan showed first signs of medullary degeneration, enlargement of the cerebrospinal fluid spaces, and incipient cerebellar atrophy. No cognitive deficits could be detected at that time. However, relatives reported the occurrence of an affective disorder preceding the movement disorder. A further clinical examination at the age of 47 revealed a marked decline in his ability to walk. In addition, his vision in his right eye had deteriorated. The left eye had already been replaced in his childhood as a result of an accident. At the age of 49, the patient was confined to a wheelchair.

Additional psychiatric symptoms appeared. These were characterized by a reduction in distance, which manifested itself in verbal derailments. From the age of 50, the patient’s ability to speak became increasingly limited. Communication took the form of partially incomplete vocalizations and crying. There was also a progressive dementia syndrome. At the present age of 55, the patient is in need of care.

The patient has no family history of similar neurodegenerative diseases. His eldest brother (II-1 of Figure 1a) was diagnosed with glioblastoma and died shortly thereafter. A sister (II-2 of Figure 1a) died at a young age and was known to have epilepsy. The youngest brother (II-9) is described as healthy. Like the rest of the family, he lives abroad in Europe. The available blood sample from him was sent at the age of 44. Results of neurological examinations are not available. The mother is healthy for her age, except for an unsteady gait that manifested itself in her late sixties. The father separated from the family at an early age and died around the age of sixty; no information is available about his state of health.

### 2.2. Genetic Findings

Repeat expansions at the common SCA loci were in the normal range (Supplementary Table S1). In particular, the repeat length of *ATXN7* (SCA7), whose expansion is associated with progressive cerebellar ataxia and retinopathy, was normal (10/12 repeats). The repeat length of *C9orf72* was also within the normal range (2/8). Similarly, the other SCA loci screened for point mutations showed no pathogenic variants.

**Figure 2:**
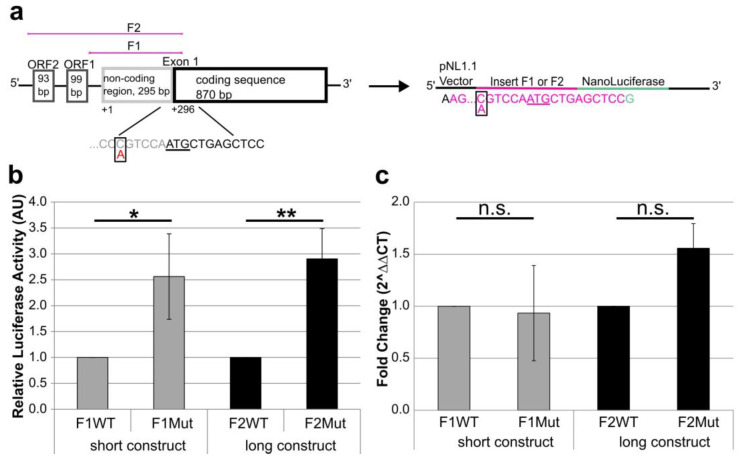
Diagram showing the structure of the 5′ region of *KCNC3*, construction of reporter plasmids and functional analysis. (**a**) The promoter region of *KCNC3* contains two ORFs of 33 amino acids (ORF1) and 31 amino acids (ORF2) (not to scale). These two ORFs are not included in the *KCNC3* transcript. Exon 1 consists of a 5′ non-coding region of 295 bp and the ATG start codon embedded in a coding sequence of 870 bp. The c.-6C>A variant is located in the extended Kozak sequence. The *KCNC3* 5′ region and the Kozak sequence have been inserted upstream of the luciferase gene. Construct F1 contains a short part of the promoter region of 363 bp and none of the ORFs. Fragment F2 spans 724 bp and contains the two ORFs. The extended Kozak sequence corresponds to either the wild-type c.-6C (WT) or the variant c.-6C>A (Mut). (**b**) Protein expression analysis of NanoLuciferase reporter. Relative luciferase activity is shown on the y-axis. NanoLuciferase activity was normalized to a constitutively expressed firefly luciferase and the corresponding wild-type construct was set to 1. The mean and standard deviation was calculated from three independent experiments (* *p* < 0.05; ** *p* < 0.01). (**c**) NanoLuciferase reporter mRNA expression analysis. Quantitative RT-PCR results are analyzed using the ∆∆CT-method. The ∆CT values are referred to the corresponding wild-type construct to obtain ∆∆CT values. Therefore, the fold change of the mutant constructs compared to the wild-type constructs is shown. The mean and standard deviation were calculated from three independent experiments (n.s., not significant).

Sequencing of the exons of *KCNC3* (SCA13; Supplementary Table S2a) revealed a non-coding variant located six bases upstream of the start codon (Figure 1b). The cytosine to adenine change (NM_004977.3:c.-6C>A; rs111909830) affects the extended Kozak sequence of *KCNC3*. The patient’s unaffected mother (Figure 1a, I-2) did not carry the variant. However, his yet unaffected younger brother (Figure 1a, II-9) did. Further family members were not available for testing.

As the index patient’s symptoms had deteriorated unexpectedly rapidly, the genetic diagnosis was extended to investigate the presence of other pathogenic variants. Analysis of 26 dementia-associated genes revealed no evidence of a genetic cause for the index patient’s disease (Supplementary Material S1). In the 136-gene ataxia panel, as well as in the whole exome, variant NM_006946.4:c.1898G>A (rs759321471) was detected in the patient’s *SPTBN2* gene. The variant leads to a change at amino acid (aa) position 633, in which arginine is replaced by glutamine (p.R633Q). The variant is rare, with a postulated minor allele frequency (MAF) <0.0001 in the alpha database release 3 [24]. Bioinformatic analysis tools such as MetaSVM release 0.5.4, MetaLR v2, and MutationTaster2 [25] classify the variant as deleterious. The given Combined Annotation Dependent Depletion (CADD)-Score [26] for p.R633Q is 23.9. Pathogenic variants in *SPTBN2* are associated with autosomal dominant SCA5 [27] and autosomal recessive spinocerebellar ataxia type 14 (SCAR14) [28]. The variant c.1898G>A was also found in both the mother and the still healthy younger brother of the index patient. Therefore, in accordance with the ACMG guidelines [29], this variant was considered to be of uncertain significance (VUS) on the basis of criteria PM1, PM2 and PP3.

Exome analysis, considering different inheritance patterns and data from the mother and brother, did not reveal any other novel candidate genes that could explain the index patient’s severe disease. This brought the *KCNC3* variant NM_004977.3:c.-6C>A (rs111909830) into focus. Variant c.-6C>A was described in genome databases once in 6448 exomes (ExAC Browser, MAF: 0.0002), in gnomAD exome browser once in 29102 exomes (MAF: 0.00003), and in gnomAD genome browser twice in 29786 genomes (MAF: 0.00007). Variant c.-6C>A has a CADD-Score of 20.2. It was classified as VUS according to the ACMG guidelines, citing the PM2 criterion.

Bioinformatic predictions for non-coding regions are limited. Programs that specialize in changes in translation start regions, such as Netstart 1.0 [30] and PreTIS v1 [31], did not predict significant differences in the altered Kozak sequence compared to the *KCNC3* wild-type. Changes in non-coding regions therefore require functional verification. Since 5′ variants in *KCNC3* have not been associated with SCA13, the possible consequences of c.-6C>A were investigated.

### 2.3. Effect of c.-6C>A on mRNA Expression and Protein Expression

A luciferase reporter assay was performed with *KCNC3* 5′-UTR fragments of different lengths, including the non-coding region of exon 1. Fragment F2 constructs (F2WT, F2Mut) contain two ORFs located in the extended promoter region and the non-coding region of exon 1. Fragment F1 constructs (F1WT, F1Mut) contain only the core promoter without ORFs and the non-coding region of exon 1 of *KCNC3* (Figure 2a).

To adapt the Kozak sequence of the luciferase gene to the *KCNC3* Kozak sequence, the ATG start codon of the luciferase reporter was replaced by the start codon of *KCNC3* and the following nine bases: CTGAGCTCC (Figure 2a). As a result, the guanine at +4 of the luciferase gene was replaced by a cytosine in all constructs because of its importance for translation efficiency. The four constructs, F1Mut, F1WT, F2Mut and F2WT, under the control of the *KCNC3* promoter were tested in a dual-luciferase assay by measuring luciferase activity. As the activity is proportional to the amount of protein, this assay provides insight into the effect of the base change on protein expression.

Despite of its short 5′ region of 363 bp, the F1 construct leads to a similar level of expression as the F2 construct of 724 bp (Supplementary Figure S1a). However, luciferase activity was significantly increased in constructs with the Kozak variant (F1Mut and F2Mut) compared to the wild-type sequence. The activity of F1Mut was 2.56 times higher than that of F1WT (156% increase), and that of F2Mut was 2.90 times higher than that of F2WT (190% increase, Figure 2b).

An increase in luciferase activity and thus protein expression can be attributed to an increase in mRNA levels. To test this hypothesis, quantitative RT-PCR experiments were performed. For comparison of mRNA levels between constructs, ∆CT values have been used as these values are normally distributed (Supplementary Figure S1b). Fold change of constructs F1Mut and F2Mut compared to constructs F1WT and F2WT, respectively, is shown in Figure 2c. Like the luciferase assay results, there was no difference in mRNA levels between the F1 wild-type construct and the F2 wild-type construct. Interestingly, there was also no significant increase in mRNA levels from wild-type to mutant constructs (Figure 2c, Supplementary Figure S1b). Taken together, these data suggest that the luciferase activity and thus protein expression of the reporter is increased by the Kozak variant, but not mRNA expression.

### 2.4. Methylation Analysis of c.-6C

**Figure 3:**
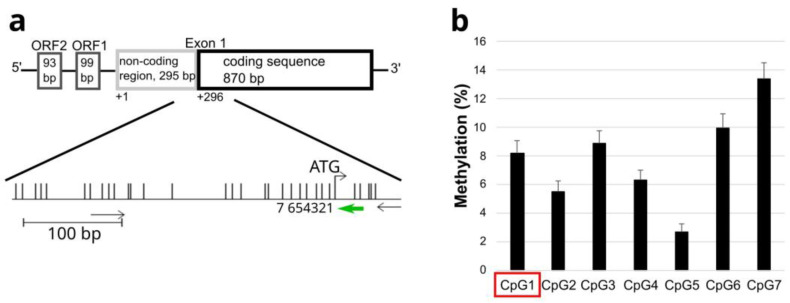
Methylation analysis of the 5′ non-coding region of *KCNC3*. (**a**) The CpG sites in the 5′ non-coding region of *KCNC3* are indicated. The graphic was generated using the python vs. cobra program (https://launchpad.net/python.vs.cobra, accessed on 20 October 2024). The translation initiation site is indicated as ATG. Seven CpG dinucleotides upstream of ATG, including the position of the c.-6C>A variant (CpG 1), were analyzed by bisulfite pyrosequencing. Black arrows below the sequence indicate the forward and reverse primers, the green arrow indicates the sequencing primer for pyrosequencing. (**b**) The methylation status of CpG 1-7 in the 5′ non-coding region of exon 1 of *KCNC3* is shown. CpG1 is framed in red, as the position of the c.-6C>A variant. The mean and standard deviation were calculated from three independent experiments.

Exon 1 of *KCNC3* is located within a large CpG island together with the promoter region (Figure 3a). The c.-6C>A variant affects not only the Kozak sequence but is also located in a CpG site in the 5′ non-coding region of *KCNC3*; therefore, the methylation level was analyzed in three wild-type controls, as sufficient genomic DNA (gDNA) of the patient was not available. Bisulfite pyrosequencing shows that wild-type c.-6C is only slightly methylated (8.17%, Figure 3b). Methylation of six additional CpG sites further upstream ranges from 2.67% to 13.38% (CpG 2: 5.5%, CpG 3: 8.89%, CpG 4: 6.28%, CpG 5: 2.67%, CpG 6: 9.94%, CpG 7: 13.38%).

In conclusion, the CpG site overlapping the Kozak sequence is poorly methylated under physiological (wild-type) conditions. Therefore, the loss of a CpG site caused by the C to A change is unlikely to have a direct effect on mRNA expression. This is also consistent with the finding that the mRNA levels are not affected by the base change. A decrease in methylation could lead to increased mRNA expression. This was not seen in the quantitative RT-PCR analysis (Figure 2c).

## 3. Discussion

In a patient with an ataxic movement disorder, two variants of uncertain significance in the genes *SPTBN2* and *KCNC3* associated with SCA were detected. The missense variant c.1898G>A in *SPTBN2* (p.R633Q in β-III spectrin) is associated with SCA5/SCAR14 [27,28]. There may be a dominant effect of the p.R633Q β-III spectrin variant, assuming an intrafamilial difference in age of onset. SCA5 is characterized by a slow progression of the disease [27], which may explain the mother’s unsteady gait in her late sixties. However, the rapid deterioration observed in the index patient within a few years cannot be caused by the *SPTBN2* variant alone. Since interactions between the gene products of several ataxia loci, including β-III spectrin and Kv3.3, are currently being investigated [32], the *KCNC3* variant c.-6C>A was further analyzed.

The novel variant c.-6C>A was located in the Kozak sequence of *KCNC3*. Pathogenic variants in *KCNC3* are associated with SCA13 [13]. They are found in the transmembrane domains and especially in the gating domain of the derived potassium ion channel Kv3.3, but less frequently in the N- and C-terminal regions [13,15,16,17,18,19,20,21,22,23]. These variants lead to either a loss of proper channel function, localization or to a disturbance in neuron excitability [13,33,34]. In contrast, the variant c.-6C>A described and studied here is localized in a cis-located regulatory element, the Kozak sequence.

Quantitative PCR on luciferase expression constructs with 5′-UTR regions of different lengths did not reveal any differences in transcript levels between wild-type and variant. The result is reasonable because neither the core promoter elements, such as the TATA and CAAT-box, nor gene-specific upstream elements are altered. Variants in core promoter elements are known to be associated with disease. For example, variants in the TATA-box lead to a mild form of β-thalassemia by reducing the expression of β-globin genes [35,36].

Upstream variants in promoter elements of the amyloid precursor protein (*APP*) gene lead to altered binding behavior of transcription factors and are associated with Alzheimer’s disease [37]. Recently, copy number variations in enhancer regions of SCA-associated genes have been investigated, but no association with the occurrence of SCA has been demonstrated [38].

Alterations at the translational start of the Kozak sequence have already been associated with various disease entities such as atrial septal defects, chronic granulomatous disease, pontocerebellar hypoplasia and others [7,39,40,41].

Common to these examples is that the Kozak sequence change leads to a reduction in translation and protein levels, resulting in haploinsufficiency as a cause of disease. In contrast to our results and others’ [8,9,41], the c.-6G>C variant in *GATA4*, which is associated with atrial septal defects, was also shown to reduce protein levels, but not mRNA levels [40]. A similar effect was shown for the Kozak variant c.-2A>G in the *RARS2* gene, which is associated with pontocerebellar hypoplasia [7].

Interestingly, both the short construct F1 and the long construct F2 containing the two small ORFs were found to have increased expression by the c.-6C>A *KCNC3* variant located in the Kozak sequence compared to the wild-type (156% for F1Mut, and 190% for F2Mut, respectively). In chronic granulomatous disease, experimental alignment of the modified Kozak sequence in *NCF1* to the Kozak consensus sequence was shown to abolish haploinsufficiency [41]. In this case, expression was increased by 50-60 % by base-editing the Kozak sequence [41]. Similarly, the Kozak variant c.-5T>C in the glycoprotein Ibα (*GPIb*α) gene has been reported to increase GPIbα expression by 15–59% [8,9], leading to thrombosis and infarction [8] and Graves’ disease [9]. Therefore, the strong overexpression by the *KCNC3* variant could lead to a cytotoxic effect.

Since the ORFs are located in the promoter region and not in exon 1, they were expected to influence the transcription rate rather than translation of *KCNC3*. The transcription levels in our experimental set do not show any changes; therefore, the increased expression must be due to increased translation initiation caused by the closer match of the c.-6C>A variant to the consensus Kozak sequence. Given its impact on protein expression, the Kozak sequence represents a key target for base editing technology [42]. In future studies, precise modulation of this sequence may be exploited for therapeutic purposes [41].

Since both overexpression and haploinsufficiency of genes can cause disease, protein levels must be tightly controlled to prevent pathogenic effects due to dosage sensitivity. *PMP22* expression can be disease-causing in both haploinsufficiency and overexpression due to gene duplication [43]. Pathogenic effects of increased ion channel function have been described for the potassium channel Kv4.3 (*KCND3*). Here, certain missense mutations increase the current density of Kv4.3 channels and are associated with cardiac electrical disorders such as Brugada syndrome and sudden unexplained death syndrome [44,45].

In recent years, translationally relevant elements have been described in the 5′-UTR, which are very short ORFs [10]. Some of these 5′-UTRs generate novel short reading frames that are associated with disease [46]. There are two small ORFs in the promoter of *KCNC3*; however, no difference in protein or mRNA expression was observed between construct F2 (ORFs) and F1 (without ORFs). This suggests that the two upstream ORFs of *KCNC3* do not affect its increased expression caused by the c.-6C>A variant.

A new mechanism for the development of SCA13 could be the overexpression of *KCNC3* by the Kozak variant c.-6C>A. Therefore, following functional analysis of c.-6C>A, the variant was reclassified as likely pathogenic according to ACMG, citing PS3 and PM2. However, it cannot be excluded that the patient has additional, as yet unidentified, genetic components leading to his disease, as exome analyses only reveal about 43% of the causes [47]. The pathogenic *KCNC3* missense variants described so far are assumed to be completely penetrant [12]. An exception is the p.S591G variant, which was found in two affected adolescents and in their healthy 65-year-old father [17]. It is speculative how the Kozak variant c.-6C>A behaves with regard to intrafamilial inheritance. A reduced penetrance cannot be ruled out, therefore the data must be interpreted with caution, particularly as no recent health data are available for the index patient’s siblings. 

Nevertheless, increased attention should be paid to variants in the regulatory elements of diseases with a suspected genetic background.

## 4. Patients and Methods

### 4.1. Patients

The index patient originates from a German–Italian family. An ataxic movement disorder was diagnosed in a hospital specialized in neurodegenerative disorders. The index patient, his youngest brother and his mother provided written informed consent for the donation of blood samples according to the guidelines of the German Genetics Diagnostics Act. The study was conducted in accordance with the principles of the Declaration of Helsinki and was approved by the Ethics Committee of the Justus Liebig University Giessen (AZ24/14_erw2019).

### 4.2. Genetic Analysis

DNA was extracted from peripheral blood samples using standard procedures. Repeat expansions at various loci (Supplementary Table S1) and pathogenic variants in the corresponding loci for SCA14, 15/16, 19, 23, 28, and 48 were initially excluded. The conditions for fragment-length analysis at the *C9orf72* locus associated with dementia have been described elsewhere [48].

The SCA13-associated gene *KCNC3* (ENSG00000131398) was analyzed by amplification and subsequent sequencing the four exons of transcript ENST00000477616.2. Primer sequences are shown in Supplementary Table S2. Subsequent exome analysis was performed. A virtual 136-gene ataxia panel was analyzed, including dominant and recessive ataxias, and relevant differential diagnoses. In addition, a dementia panel consisting of 26 genes was analyzed (Supplementary Material S1). Finally, a subtractive trio exome including the index patient, the healthy mother (I-2 of Figure 1a) and a yet unaffected brother (II-9 of Figure 1a) was analyzed. For enrichment, the SureSelect XT HS Human All Exon V8 kit (Agilent, Santa Clara, CA, USA) was used. Sequencing was performed on an Illumina NovaSeq platform (Illumina, San Diego, CA, USA). In all three DNA-samples, the mean coverage of exons was at least >20× with >98.8%, and >50× with 89.6% targeted bases covered. Filter criteria for likely pathogenic variants were MAF <0.01 in the gnomAD v2.1.1 database [49], HPO terms and OMIM entry. Different inheritances as well as de novo mutations were considered in the analysis. The reference genome was GRCh37. Varvis software v.1.24.1 (Limbus Medical Technologies GmbH, Rostock, Germany) was used for analysis of single nucleotide variants (SNVs) and copy number variants (CNVs).

### 4.3. Plasmid Constructs

To clone the Kozak sequence and the extended 5‘ region of *KCNC3*, four different primers were used to create the following constructs: Forward primer KCNC3-F2-HindIII (5′-ACCAAGCTTCACCTTCTCACCGAGTCTAAGTC-3′) was used either with reverse primer KCNC3-WT-NcoI-rev derived from the wild-type sequence (5′-TTTCCATGGGAGCTCAGCATTGGACGGGGGGC-3′), or primer KCNC3-Mut-NcoI-rev derived from the mutant sequence (5′-TTTCCATGGGAGCTCAGCATTGGACTGGGGGC-3′) to amplify a 747 bp fragment on genomic DNA. Primer KCNC3-F1-HindIII (5‘-ACCAAGCTTGTCTCTCTCTATCGTATCTAGCCC-3‘) was used in combination with the reverse primers above to produce a shorter fragment (386 bp). Primers are listed in Supplementary Table S2b. Forward primer also encoded the HindIII restriction site and reverse primer encoded the NcoI restriction site at their 5‘-ends (underlined). After amplification, fragments were restricted with HindIII and NcoI (New England Biolabs, Ipswich, MA, USA) and cloned in vector pNL1.1 (Promega GmbH, Madison, WI, USA) upstream of the NanoLuciferase reporter. The ATG start codon of the luciferase gene is therefore replaced by the *KCNC3* start codon and its nine downstream bases.

Competent JM109 *E. coli* cells (Promega GmbH, Madison, WI, USA) were transformed with the recombinant plasmids. Integrity of the selected clones was confirmed by sequencing. To maintain the reading frame of the luciferase gene, plasmids were again restricted with NcoI and subsequently digested with Mung Bean Nuclease (New England Biolabs, Ipswich, MA, USA). After transforming *E. coli* cells with the resulting plasmids, the correct clones were selected by sequencing and used for transfection.

### 4.4. Cell Culture and Luciferase Assay

Human embryonic kidney 293T (HEK 293T, American Type Culture Collection, Manassas, VA, USA) cells were cultured in minimal Eagle’s medium (MEM, Gibco, Waltham, MA, USA), supplemented with 10% fetal bovine serum (FBS, Sigma-Aldrich, St. Louis, MO, USA) and 1% Penicillin/Streptomycin (Gibco, Waltham, MA, USA). Cells were seeded at 4 × 10^5^ cells per well in 6 well plates. After 24 h, cells were transfected with 2 µg of target NanoLuciferase construct and 1 µg of control firefly luciferase plasmid pGL4.54 (Promega GmbH, Madison, WI, USA) in a total volume of 150 µL of Opti-MEM (Gibco, Waltham, MA, USA) and 7.5 µL of FUGENE HD (Promega GmbH, Madison, WI, USA), following the manufacturer’s instructions. For quantitative real-time (RT)-PCR experiments, 1 µg of target NanoLuciferase construct together with 2.5 µL FUGENE HD in a total volume of 50 µL were transfected per well. Cells were washed with phosphate buffered saline (PBS) and lysed in 200 µL of passive lysis buffer (Promega GmbH, Madison, WI, USA) 24 h after transfection. 80 µL cell lysate was added to the Nano-Glo^®^ Dual-Luciferase^®^ Reporter Assay System (Promega GmbH, Madison, WI, USA). The assay was performed according to manufacturer’s instructions in white 96 well plates (Corning Inc., Kennebunk, ME, USA); measurements were performed on the Orion L Microplate Luminometer (Berthold Technologies GmbH & Co. KG, Bad Wildbad, Germany). The experiments were carried out as biological triplicate and each as technical triplicate. The results were analyzed by two-sided unpaired *t*-test in Excel (Microsoft Cooperation, Redmond, WA, USA).

### 4.5. Quantitative RT-PCR

28 h after transfection, HEK 293T cells were harvested and washed with PBS. Pellets were stored at −80 °C. RNA preparation was performed with Monarch Total RNA Miniprep Kit (New England Biolabs, Ipswich, MA, USA). RNA concentration was determined on a NP80 Nanodrop (Implen GmbH, München) and samples’ A260/A280 and A260/A230 ratios were >2. To minimize residual plasmid DNA content, 1 µg of RNA was additionally treated with DNAseI (Invitrogen, Waltham, MA, USA). Afterwards, 100 ng of treated total RNA was reversely transcribed with SuperScriptIII (Invitrogen, Waltham, MA, USA). *NanoLuciferase* and *ACTB* endpoint PCRs were performed with corresponding primers to confirm reverse transcription. Quantitative RT-PCR was performed on a CFX384 cycler (Bio-Rad Laboratories, Hercules, CA, USA) with iTaq Universal SYBRgreen Supermix (Bio-Rad). Primers used to detect *NanoLuciferase*, *ACTB*, *GAPDH* and *TBP* are listed in Supplementary Table S2c. The relative transcript levels of *NanoLuciferase* compared to the wild-type constructs were calculated by the ∆∆CT-method. For *ACTB*, *TBP* and *GAPDH*, the average of all CT values was calculated and used as an internal control. The experiments were performed in three biological replicates and in each case in technical duplicates. Statistical analysis was performed by two-sided unpaired *t*-test on ∆CT values (CT(*internal control*)-CT(*NanoLuciferase*)).

### 4.6. Methylation Analysis

Methylation analysis was performed by bisulfite pyrosequencing. Genomic DNA of three healthy controls (two males, one female) was extracted from peripheral blood samples using standard procedures. 1500 ng or 500 ng gDNA was converted with Epitect Bisulfite Kit (Qiagen GmbH, Hilden, Germany). Bisulfite converted DNA was amplified with Epimark Taq Polymerase (New England Biolabs, Ipswich, USA), using reverse primer KCNC3_BS_Rev and forward primer KCNC3_BS_Fw_bio, respectively (Supplementary Table S2d). Pyrosequencing was performed according to the manufacturer’s guidelines using the PyroMark Q24 System (Qiagen) and sequencing primer KCNC3_BS_revSeq. The experiment was performed with three independent bisulfite reactions for each sample and the average methylation frequency was calculated for each CpG site.

## Data Availability

Data generated during this study are included in this manuscript and in Supplementary Files. Material (plasmids) are available on request from the authors.

## Supplementary Materials

The following supporting information can be downloaded at: https://www.mdpi.com/article/10.3390/ijms252212444/s1.
