# Supplementary material for: Kv3.3 Expression Enhanced by a Novel Variant in the Kozak Sequence of KCNC3
Source: Int J Mol Sci. 2024 Nov 20;25(22):12444. doi: 10.3390/ijms252212444 (PMC11595341; doi:10.3390/ijms252212444)
Supplement: Supplementary file 1 [file ijms-25-12444-s001.zip › ijms-3306340-supplementary.pdf]

Supplementary Materials

International Journal of Molecular Sciences

Kv3.3 expression enhanced by a novel variant in the Kozak sequence of *KCNC3*

Marlen Colleen Reis[1], Frauke Härtel[1,2], Antje Maria Richter[3], Michaela Weiß[1], Lea-Theresa Mösle[1,4], Reinhard Dammann[3], Dagmar Nolte[1]

1 Institute of Human Genetics, Justus Liebig University Giessen, Germany
2 present address: Institute of Physiology, Justus Liebig University Giessen, Germany
3 Institute of Genetics, Justus Liebig University Giessen, Germany
4 present address: Innere medizinische Klinik II, Klinikum Memmingen, Germany

Corresponding author:
Dagmar Nolte, Institute of Human Genetics, Justus Liebig University Giessen
Schlangenzahl 14, 35392 Giessen, Germany.
Tel.: +49-641-9941615, FAX: +49-641-9941609
dagmar.nolte@humangenetik.med.uni-giessen.de
ORCID: 0000-0001-5710-8790

Supplementary Table S1: Index patient allele size for common repeat expansion diseases

**Disorder**	**Gene**	**Inheritance**	**Allele sizes [Repeats]**
SCA1	*ATXN1*	autosomal-dominant	31/31
SCA2	*ATXN2*	autosomal-dominant	22/22
SCA3	*ATXN3*	autosomal-dominant	21/31
SCA6	*CACNA1A*	autosomal-dominant	11/13
SCA7	*ATXN7*	autosomal-dominant	10/12
SCA8	*ATXN8*	autosomal-dominant	23/29
SCA12	*PPP2R2B*	autosomal-dominant	10/10
SCA17	*TBP*	autosomal-dominant	36/37
SCA27B	*FGF14*	autosomal-dominant	59/69
Frontotemporal dementia/ ALS	*C9orf72*	autosomal-dominant	2/8
CANVAS	*RFC1*	autosomal-recessive	ca. 11/11*

SCA: spinocerebellar ataxia; ALS: amyotrophic lateral sclerosis; CANVAS: Cerebellar ataxia, neuropathy and vestibular areflexia syndrome; * one normal sized allele was detected

Supplementary Table S2: Primer sequences for *KCNC3* sequencing, promoter construct cloning, quantitative RT-PCR, and methylation analysis

Primer	Sequence 5'->3'	Application
**a**		*KCNC3* Sanger Sequencing
Ex1_F	CAGCTGGGTCGCGTTAGAGTG	
Ex1_R	CAGGCTGCTGCTGCTGCGG	
Ex1_6F	CGGCTTCTGGGGCATCGACG	
Ex1_7R	GGGTTGGAGCTGGGGCGCT	
Ex1_8F	GCGCCACCGCCGCAGCCG	
Ex1_9R	GTCGGGCGCCTCGAAGGAGTC	
Ex2_F	GGGCACTGGAAGGGTCTTCTGG	
Ex2_R	CTCACCTCTTCGACGCCAACCAC	
Ex3_F	CCCTTGAATCTCCACTTCCAGT	
Ex4_R	GGTTAACTGGGTGCAGCTGCT	
**b**		*KCNC3*- promoter construct cloning
KCNC3-F1-HindIII	ACCAAGCTTGTCTCTCTCTATC GTATCTAGCCC	Amplification of fragment F1 of *KCNC3* promoter
KCNC3-F2-HindIII	ACCAAGCTTCACCTTCTCACCG AGTCTAAGTC	Amplification of fragment F2 of *KCNC3* promoter
KCNC3-WT-NcoI-rev	TTTCCATGGGAGCTCAGCATTG GACGGGGGGC	Amplification of *KCNC3* start codon with wild-type Kozak sequence
KCNC3-Mut-NcoI-rev	TTTCCATGGGAGCTCAGCATTG GACTGGGGGC	Amplification of *KCNC3* start codon with mutated Kozak sequence
**c**		quantitative PCR (qPCR)
NanoLuc_F	TGGTGTACCCTGTGGATGAT	Detection of NanoLuciferase
NanoLuc_R	CTCCGTTGATGGTTACTCGG	
qTBP_cDNA_F	GTGAATCTTGGTTGTAAACTTGAC	*TBP* qPCR, reference gene
qTBP_cDNA_R	CAATTCTGGGTTTGATCATTCTG	
ACTB_Fw	GGACTTCGAGCAAGAGATGG	*ACTB* qPCR, reference gene
ACTB_Rv	AGCACTGTGTTGGCGTACAG	
GAPD_Fw	GAGTCAACGGATTTGGTCGT	*GAPDH* qPCR, reference gene
GAPD_Rv	TTGATTTTGGAGGGATCTCG	
**d**		Pyrosequencing
KCNC3_BS_Fw_bio	GTTTTATTGGYGGTTTYGATTYG TTTAGTTGGG	
KCNC3_BS_Rev	ACTACTACTTACTAACCCCCTAA	
KCNC3_BS_revSeq	AAACRCAAACTAAACTCAA	Sequencing primer

Supplementary Material S1:

Panels of ataxia and dementia genes

Genes included in panel 'SCA/SCAR/differential diagnosis - all genes':

ABCB7, ABHD12, AFG3L2, ANO10, APTX, ARSA, ATCAY, ATG7, ATM, ATP1A3, ATP8A2, ATXN1, ATXN2, ATXN3, ATXN7, ATXN10, CA8, CACNA1A, CACNA1G, CACNB4, CAMTA1, CAPN1, CCDC88C, CLCN2, CLN6, COA7, COQ8A, CP, CTBP1, CWF19L1, CYP7B1, CYP27A1, DAB1, DARS2, DDHD2, DNAJC5, DNMT1, DOOH, EIF2B1, EIF2B2, EIF2B3, EIF2B4, EIF2B5, ELOVL4, ELOVL5, EPM2A, EXOSC5, FAT2, FGF14, FLVCR1, FXN, GBA2, GDAP2, GFAP, GOSR2, GRID2, GRM1, HEXA, HEXB, HSD17B4, ITPR1, KCNA1, KCNC3, KCND3, KCNJ10, KCNN2, KIF1A, KIF1B, KIF1C, LCN2, MARS2, MRE11, MTTP, NKX6-2, NOP56, NPC1, NPC2, PDYN, PEX7, PHYH, PIK3R5, PITRM1, PLA2G6, PLD3, PMPCA, PNKP, PNPLA6, POLG, POLR3A, POU4F1, PRICKLE1, PPP2R2B, PRKCG, PRRT2, PUM1, RFC1, RNF170, RNF216, RUBCN, SACS, SAMD9L, SCN2A, SCYL1, SETX, SIL1, SLC1A3, SLC2A1, SLC52A2, SLC52A3, SLC9A1, SNX14, SOD1, SPG7, SPTBN2, STUB1, SYNE1, TBP, TDP1, TDP2, TGM6, THG1L, TMEM240, TPP1, TTBK2, TTC19, TTPA, TWNK, TUBB4A, VAMP1, VLDLR, VPS13D, VPS41, WDR81, WFS1, WWOX, XRCC1

Genes included in panel 'early-onset dementia/differential diagnoses':

APP, c9orf72, CHCHD10, CHMP2B, CSF1R, GBA1, GRN, ITM2B, MAPT, MPO, NOTCH3, NOS3, OPTN, PLAU, PRNP, PSEN1, PSEN2, SNCA, SNCB, SQSTM1, TARDBP, TBK1, TREM2, TRPM7, UBQLN2, VCP

Supplementary Figure S1

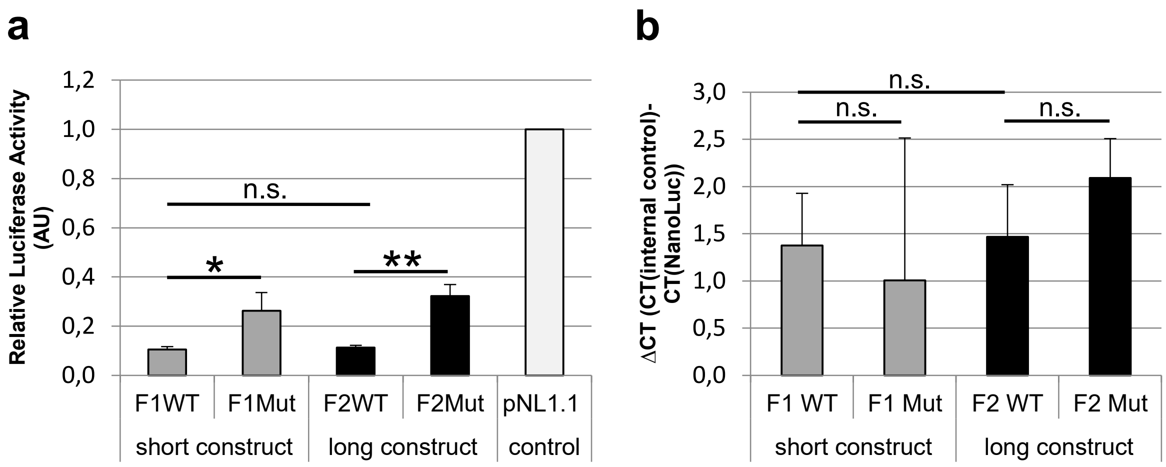

(a) Protein expression analysis of NanoLuciferase reporter. Relative luciferase activity is given on the y-axis. NanoLuciferase activity was normalized to a constitutive expressed firefly luciferase and a pNL1.1 control vector was set to 1 to compare F1WT and F2WT constructs. Mean and standard deviation was calculated from three independent experiments ($p<0.05$ *; $p<0.01$ **; n.s. not significant). (b) mRNA expression analysis of NanoLuciferase reporter. Quantitative RT-PCR results are analyzed with the ΔΔ CT-method. ΔCT values are shown. Mean and standard deviation was calculated from three independent experiments.
